# Sequentially Integrated Optimization of the Conditions to Obtain a High-Protein and Low-Antinutritional Factors Protein Isolate from Edible *Jatropha curcas* Seed Cake

**DOI:** 10.5402/2013/197201

**Published:** 2013-02-13

**Authors:** Liliana León-López, Gloria Dávila-Ortiz, Cristian Jiménez-Martínez, Humberto Hernández-Sánchez

**Affiliations:** Departamento de Graduados e Investigación en Alimentos, Escuela Nacional de Ciencias Biológicas, Instituto Politécnico Nacional, Carpio y Plan de Ayala, 11340 México, DF, Mexico

## Abstract

*Jatropha curcas* seed cake is a protein-rich byproduct of oil extraction which could be used to produce protein isolates. The purpose of this study was the optimization of the protein isolation process from the seed cake of an edible provenance of *J. curcas* by an alkaline extraction followed by isoelectric precipitation method via a sequentially integrated optimization approach. The influence of four different factors (solubilization pH, extraction temperature, NaCl addition, and precipitation pH) on the protein and antinutritional compounds content of the isolate was evaluated. The estimated optimal conditions were an extraction temperature of 20°C, a precipitation pH of 4, and an amount of NaCl in the extraction solution of 0.6 M for a predicted protein content of 93.3%. Under these conditions, it was possible to obtain experimentally a protein isolate with 93.21% of proteins, 316.5 mg 100 g^−1^ of total phenolics, 2891.84 mg 100 g^−1^ of phytates and 168 mg 100 g^−1^ of saponins. The protein content of the this isolate was higher than the content reported by other authors.

## 1. Introduction

*Jatropha curcas* is a tree of the Euphorbiaceae family which has been used, due to the high oil content (40–60%) of its seeds, as an alternative source of biodiesel [1]. The residual seed cake is a low-value byproduct left after oil extraction which, however, has a high protein content [2]. This seed cake, also, is highly toxic to a number of animal species due to the presence of different types of antinutritional components such as phytic acid, trypsin inhibitors, phenolic compounds, lectins (curcin), and saponins in high amounts [3, 4]. In addition to these, phorbol esters have been identified as one of the main compounds responsible for *J. curcas* toxicity [5]. These compounds are referred to as tigliane diterpenes in which two hydroxyl groups are esterified to fatty acids and are well known for their tumor promoting activity [6]. However, edible or nontoxic provenances have been reported to exist in Mexico [3, 7] which contain negligible amounts of phorbol esters though the levels of the other antinutritional compounds are similar to those found in the toxic varieties [8]. This would allow the seed meal from edible varieties to be processed and used as an economic source of protein for both humans and animals. Plant protein isolates are very important in the food industry due to their high protein contents, which can reach 90%. They are commonly prepared from oilseeds, legumes, and their defatted seed meals. The methods of preparation generally include the solubilization of proteins in basic solutions (pH 8–11) followed by precipitation using different techniques [1]. Acid precipitation at the isoelectric point, which usually ranges from pH 4.5 to 5.0, is the general method for plant protein isolation [9]. Nevertheless, there is a need to analyze and optimize these methods [10] in order to have an optimum protein recovery from *J. curcas* seed cake that allows the minimization of the content of the antinutritional compounds mentioned before to improve the quality of the protein obtained. 

 Response surface methodology (RSM) and Taguchi's orthogonal array method are well-known optimization techniques which allow the building of an experimental design with the smallest number of experimental runs [11]. RSM is a collection of statistical and mathematical techniques used for modeling, analyzing, and optimizing problems in science and engineering [12]. There are many reports on the use of RSM for the optimization of biotechnology processes [13, 14], food processes [15], phenolic compound extraction [16], and protein precipitation methods [10]. The Taguchi method uses orthogonal arrays to study a large number of variables with a small number of experimental runs and provides information about all the parameters that affect the responses [17]. This method has been used in several fields including the chemical [17], biotechnological [18], and food industries [19]. The effectiveness and improvement of a sequential integration of the Taguchi method and RSM (TM-RSM) has been previously demonstrated [11]. The hybrid methodology compensates the weaknesses of the two techniques. The Taguchi method is not able to find the real optimum value and the RSM even with the best experimental designs uses many runs when the initial number of variables is high. In the sequentially integrated approach, the Taguchi method is used to screen and optimize the qualitative and discrete factors and the RSM uses these results in a new experimental design to model and optimize the quantitative and continuous variables with more accuracy to produce better solutions.

The purpose of this work was then to optimize the protein isolation process from *J. curcas* L. seed cake by the isoelectric precipitation method via a sequentially integrated optimization approach (TM-RSM). The influence of four different factors (solubilization pH, extraction temperature, NaCl addition, and precipitation pH) on the protein and antinutritional compounds content of the isolate will be analyzed.

## 2. Materials and Methods

### 2.1. Preparation and Analysis of Raw Material

The edible *J. curcas* seeds used in this study were obtained from ripe fruits harvested in Yautepec, Morelos, Mexico. The whole seeds (kernels plus shells) were partially defatted by mechanical pressing. The seed cake obtained was milled using a kitchen blender (Oster 10-speed blender, Osterizer). The residual oil present in this flour was removed using hexane (boiling point 60°C) in a Soxhlet apparatus for 10 h. The defatted flour was dried and finally passed through a sieve (20-mesh screen). 

The crude protein (*N* × 6.25) content of *J. curcas* seeds, seed cake, flour, and isolates was determined according to the standard methods of the Association of Official Analytical Chemists [20]. All analyses were performed in triplicate.

### 2.2. Preparation of Protein Isolates

The protein isolates were prepared with the different conditions indicated in the experimental designs following the general scheme of Makkar et al. [21]. The flour was suspended in distilled water (1 : 10) with or without NaCl and the suspension was adjusted to alkaline pH with 1 N NaOH and stirred at constant temperature for 1 h.

The suspension was then centrifuged at 18000 ×g for 30 min at 15°C. The supernatant was filtered and collected. The pH of this solution was adjusted with 1 N HCl for protein precipitation. The precipitated proteins were collected by centrifugation (18000 ×g, 30 min, 4°C) and freeze-dried. 

### 2.3. Determination of Toxic Compounds and Antinutritional Factors

Total phenolics were extracted using acidified methanol and quantified by the Folin-Ciocalteu reagent [22] and expressed as tannic acid equivalents 100 g^−1^. The phytic acid content of the samples was determined by the colorimetric procedure described by Vaintraub and Lapteva [23]. Suitable aliquots were diluted with distilled water to make 3 mL and then used for the assay. Results were expressed as mg 100 g^−1^ phytic acid, using as a standard phytic acid (sodium salt; Sigma, St. Louis, MO, USA) [4]. Total saponin content was determined using the spectrophotometric method described by Hiai et al. [24]. The concentration of saponins was interpolated from a standard curve of different concentrations of diosgenin in 80% aqueous methanol and expressed as diosgenin equivalents 100 g^−1^ [4].

### 2.4. Experimental Design and Statistical Analysis

There are many operational variables which can affect the protein extraction during protein isolates preparation; therefore, only the four more influential factors were chosen: solubilization pH, extraction temperature, NaCl addition, and precipitation pH. The levels of the factors were taken from literature [21]. Based on the Taguchi method, the L9-orthogonal array was constructed as shown in Table 1. 

The protein and total phenolics content (%) of the isolates were considered as the response variables. The ANOVA procedure was used to determine the percentage of contribution of each factor to the responses and to model the relationship between these factors and the selected response. The most important factors were selected to perform a second experimental design, a three-level Box-Behnken response surface design for the optimization of the protein extraction technique. Experimental factors and levels are listed in Table 3. All results from experimental designs were analyzed using the software Design Expert 7.0.3 (Stat-Ease, Inc., USA).

## 3. Results and Discussion

### 3.1. The Taguchi Experimental Design

First, in order to identify the significant influence of each factor tested on the response variables, an ANOVA was performed with the results from the Taguchi design shown in Table 1. The influence of solubilization pH, extraction temperature, precipitation pH, and NaCl concentration of the extraction solution on the protein and phenolics content of the isolates is shown in Table 2. Extraction temperature and precipitation pH were the parameters with the strongest influence on the protein content of the isolates. As reported, appropriate heat treatments might partially break down hydrogen and disulfide bonds, resulting in an improvement in protein dissolution rate [25] and leading to obtain an isolate with higher protein content when temperature is increased. 

It has been reported that extraction in the presence of NaCl has improved the extractability of proteins from some seeds [26, 27]. Our results show that, unlike other seeds, the protein recovery decreased when NaCl is added to the extraction solution at the range tested (0.3 and 0.6 mol L^−1^) by a salting-out effect on the *Jatropha* proteins. 

Similar results were also reported by Makkar et al. [21]. However, the NaCl addition in the extraction solution has no considerable impact on the final protein content of protein isolates (0.10% contribution). Conversely, the total phenolics content was significantly and adversely affected by NaCl concentration in the solution extraction and almost negligibly by the precipitation pH.

Temperature, precipitation pH, and NaCl addition were then selected as independent variables and solubilisation pH was set constant to an 11 value in all experiments for the RSM. The three levels of these variables were the same as those used in the L9-orthogonal array performed previously. Protein, total phenolics, phytate, and saponin content, of the isolates were taken as response variables in a Box-Behnken 3^3^ experimental design. Experimental results are shown in Table 3.

The responses obtained from each run of the experimental design were analyzed by multiple regression analysis using the Design Expert software (Stat-Ease, Inc., USA) to obtain empirical models for each response. The quality of the fit of the obtained model equations was expressed by the determination coefficient *R* ^2^ and the significance of the models and their coefficients was evaluated by one-way ANOVA and Student's *t*-tests, respectively.

### 3.2. Box-Behnken Design

#### 3.2.1. Protein Content of Protein Isolates

A quadratic equation was established on the basis of analysis of the Box-Behnken experimental data as follows:

(1) Protein  content=+359.50093−0.41282∗T−116.19415∗pH+7.69070∗NaCl−0.26063∗T∗NaCl+6.79515E−003∗T2+12.67662∗pH2.

Extraction temperature and precipitation pH were the parameters that affected more strongly the protein content of the isolates. As stated above, appropriate heat treatments might partially break down hydrogen and disulfide bonds, resulting in an improvement in protein dissolution rate [25] which leads to a protein isolate with higher protein content when temperature is increased. 

**Figure 1:**
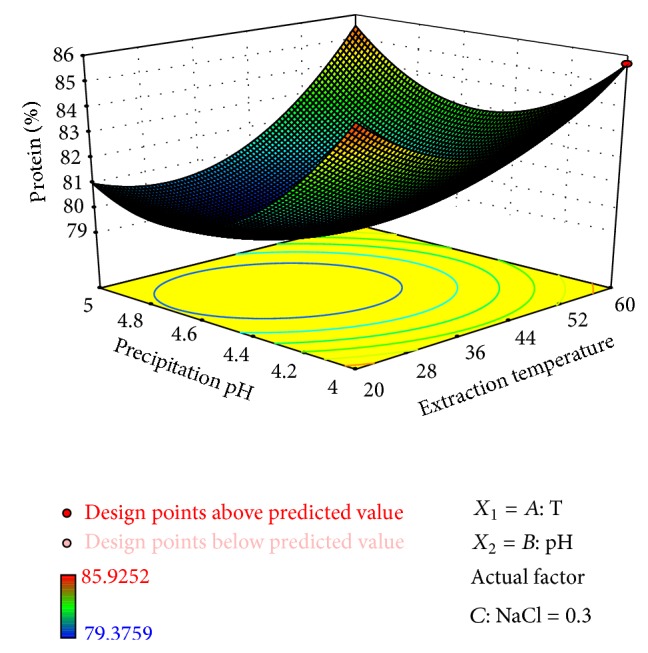
Surface plot of the protein content (%) of the protein isolate as affected by the process variables precipitation pH and extraction temperature (°C). NaCl concentration was set to 0.30 M.

On the other hand, the highest protein content was obtained by performing precipitation at pH 4. Saetae et al. [1] tested the extraction pH of *J. curcas* proteins and found the lowest yields at a pH range of 4.0 to 4.3; these results suggest that isoelectric point of most *Jatropha* proteins is very close or equal to 4; thus, a substantial amount of the protein solubilised at the alkaline pH is recovered when precipitation is performed at pH 4 (Figure 1).

#### 3.2.2. Total Phenolics

**Figure 2:**
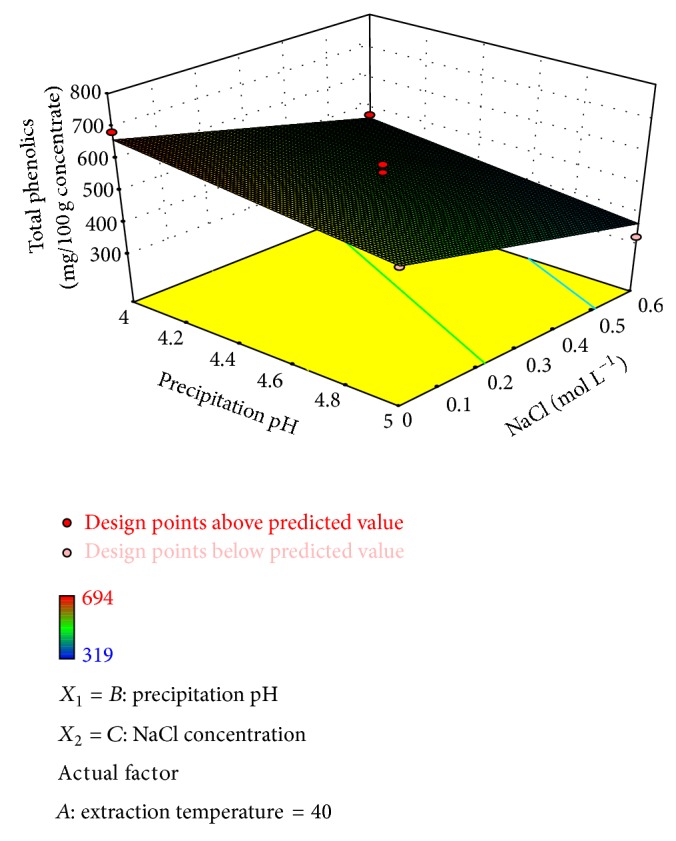
Surface plot of the total phenolics content of the protein isolate as affected by the process variables precipitation pH and NaCl concentration (temperature = 40°C).

Some of the protein isolates were dark brown in color due to shell pigments that were solubilised and precipitated along with the proteins. The use of NaCl increased the lightness of isolates in relation to those obtained by extraction with water. Moure et al. [28] observed the same behavior when testing the extraction and isolation of proteins from defatted *Gevuina avellana* seeds. The content of total phenolics ranged from 319 to 694 mg 100 g^−1^ of protein isolate. This response was significantly affected by NaCl concentration in the solution extraction and slightly by precipitation pH (64.86 and 9.88% of contribution, resp.).  Figure 2 shows the surface plots for the effect of both independent variables on the total phenolics content of protein isolates. It is observed that the content of these compounds decreased to a minimum point when the maximum concentration of NaCl was added.

 The effect of extraction temperature *per se* can be considered negligible compared to the effect of the other factors. However, there is an important effect of the interactions between extraction temperature and precipitation pH as well extraction temperature and NaCl concentration. 

#### 3.2.3. Phytates

**Figure 3:**
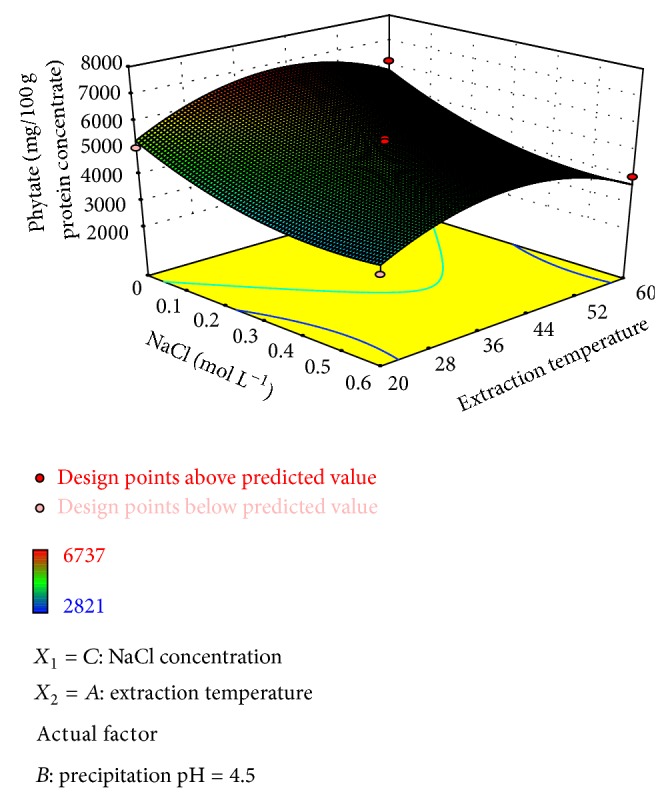
Surface plot of the phytate content of the protein isolate as affected by the process variables NaCl concentration and extraction temperature (precipitation pH = 4.5).

The analysis of the desirability function [12] showed multiple combinations of factors that allowed a minimization of the phytate content of protein isolates when extraction is performed at 60°C and NaCl is added (0.4 M) (Figure 3). This effect may be due to activation of endogenous phytases and acid phosphatases by effect of temperature. These enzymes mediate the phytates release, allowing reactions between these and NaCl present in the solution extraction to form their respective sodium salts. Cigala et al. [29] carried out solubility measurements of dodecasodium phytate in pure water and in NaCl solutions at different ionic strengths and found that the phytate solubility decreases drastically with an increase in ionic strength. So, the decrease of phytate content in the protein isolates carried out in NaCl solution could be explained by a salting-out effect on the phytate salts formed during the solubilization which are discarded along with other nonsoluble compounds by centrifugation. 

#### 3.2.4. Saponins

Alkaline solutions were used for protein extraction, and under these conditions, saponins are converted into sodium salts that are well soluble in water [30]. By analyzing the ANOVA results, the precipitation pH appeared to have a negligible effect; however, it plays an important role since the acidification of the alkaline extract, in order to precipitate proteins, converts the saponins into water-insoluble forms [30] which precipitate along with protein. The interaction of temperature and precipitation pH and the interaction of precipitation pH and NaCl concentration have the strongest influence on the content of saponins of the protein isolates. 

**Figure 4:**
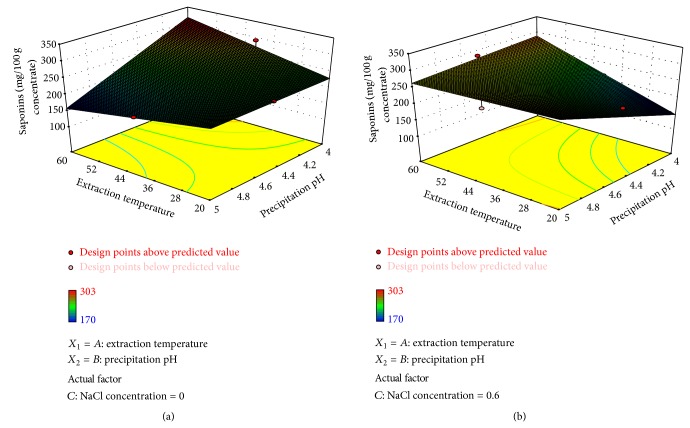
Surface plots of saponin content of protein isolates as affected by the process variables extraction temperature and precipitation pH (a) using water as extraction solution and (b) adding NaCl (0.6 M) to the solution.

The protein isolates obtained using pure water as extraction solution have the highest amount of saponins when the highest temperature is tested and the subsequent acid precipitation is carried out at pH 4 (Figure 4). Using the same conditions of temperature and extraction solution is possible to minimize the content of saponins just by performing the precipitation step at pH 5. In contrast, when NaCl is added into the extraction solution at the maximum ionic strength (0.6 M), the saponin content in the protein isolates decreases drastically when the extraction is performed at 20°C and precipitation at pH 4, and this decrease is directly proportional to the temperature extraction and the precipitation pH (Figure 4(b)).

### 3.3. Optimization of the Conditions for a High-Protein and Low-Antinutritional Factors Protein Isolate

The conditions for obtaining a *J. curcas* protein isolate from the seed cake depended strongly on the extraction temperature, NaCl concentration, and precipitation pH and were determined by RSM. The optimal values obtained, in order to maximize the protein content and minimize, at the same time, the antinutritional factors content were solubilization at 20°C into a solution of NaCl 0.6 M and precipitation at pH 4. Under these conditions, it was possible to obtain a protein isolate with 93.21% of proteins, 316.5 mg 100 g^−1^ of total phenolics, 2891.84 mg 100 g^−1^ of phytates, and 168 mg 100 g^−1^ of saponins, differing just slightly from the predicted values by 0.30% (93.3%), 0.27% (317.36), 13.13% (3327.99), and 14.17% (146.76), respectively. The use of the sequentially integrated optimization allowed us to produce a *J. curcas* isolate with a higher protein content (93.21%) than previously reported. Other authors reported protein contents of 90.1, 89, and 82% in their products [1, 2, 21]. 

## 4. Conclusion

In this work, a sequentially integrated approach was successfully applied to the optimization of conditions to prepare a *Jatropha curcas* protein isolate. The Taguchi method was used to screen four factors involved in obtaining protein isolates. Response surface methodology using a 3^3^ Box-Behnken design was used as the optimization tool for attaining the conditions for a high-protein and low-antinutritional factors (phytic, saponin, and phenolic compounds) protein isolate from edible *J. curcas* seed cake. The estimated optimal conditions were an extraction temperature of 20°C, a precipitation pH of 4, and an amount of NaCl in the extraction solution of 0.6 M. By varying the conditions of preparation, isolates with different concentrations of protein and antinutritional factors can be obtained. The addition of salts influenced the amount of antinutritional factors of the protein products. The protein content of the isolate was higher than that of other reported protein concentrates or isolates.

## Figures and Tables

**Table 1:** L9-orthogonal array and response values.

Run	Extraction temperature (°C)	Solubilisation pH	[NaCl] (mol L^−1^)	Precipitation pH	Protein (g 100 g^−1^ isolate)	Total phenolics (mg 100 g^−1^ sample)
1	20	9	0	4	78	622
2	40	10	0	5	92	596
3	60	11	0	4.5	84	502
4	60	9	0.6	5	93	335
5	20	10	0.6	4.5	75	382
6	60	10	0.3	4	89	537
7	40	11	0.6	4	87	338
8	40	9	0.3	4.5	74	510
9	60	11	0.3	5	93	443

**Table 2:** Analysis of variance of the results of the Taguchi design.

Factor	% Contribution of factor towards responses	
	Protein concentration in isolates	Total phenolics content in isolates
Solubilisation pH	6.85	2.51
Temperature	75.30	2.85
Precipitation pH	17.75	0.19
NaCl concentration	0.10	95.45

**Table 3:** Box-Behnken response surface design and response results.

Extraction temperature (°C)	[NaCl] (mol L^− 1^)	Precipitation pH	Protein (g 100 g^−1^ isolate)	Total phenolics (mg 100 g^−1^ sample)	Phytate (mg 100 g^−1^ sample)	Saponins (mg 100 g^−1^ sample)
40	4.5	0.3	87.13	546	5218	248
40	4.5	0.3	87.07	500	5229	244
40	4.0	0.0	89.89	685	6737	293
20	4.5	0.6	87.75	405	3346	239
60	4.5	0.6	87.58	452	4019	284
60	4.0	0.3	93.96	636	3395	303
40	5.0	0.0	89.62	566	5458	196
40	5.0	0.6	87.69	319	4731	250
20	4.5	0.0	87.86	694	5025	231
40	4.5	0.3	87.04	571	5308	245
60	5.0	0.3	93.41	379	2821	230
20	4.0	0.3	94.22	435	4626	170
40	4.0	0.6	91.73	467	3878	213
40	4.5	0.3	87.15	547	5431	243
20	5.0	0.3	96.69	379	2963	172
60	4.5	0.0	93.94	517	6170	221
40	4.5	0.3	87.18	441	4933	216
